# Photonic Crystal Structures for Photovoltaic Applications

**DOI:** 10.3390/ma17051196

**Published:** 2024-03-04

**Authors:** Anna Starczewska, Mirosława Kępińska

**Affiliations:** Institute of Physics—Center for Science and Education, Silesian University of Technology, Krasińskiego 8, 40-019 Katowice, Poland; miroslawa.kepinska@polsl.pl

**Keywords:** photonic crystal, photovoltaic, solar cell

## Abstract

Photonic crystals are artificial structures with a spatial periodicity of dielectric permittivity on the wavelength scale. This feature results in a spectral region over which no light can propagate within such a material, known as the photonic band gap (PBG). It leads to a unique interaction between light and matter. A photonic crystal can redirect, concentrate, or even trap incident light. Different materials (dielectrics, semiconductors, metals, polymers, etc.) and 1D, 2D, and 3D architectures (layers, inverse opal, woodpile, etc.) of photonic crystals enable great flexibility in designing the optical response of the material. This opens an extensive range of applications, including photovoltaics. Photonic crystals can be used as anti-reflective and light-trapping surfaces, back reflectors, spectrum splitters, absorption enhancers, radiation coolers, or electron transport layers. This paper presents an overview of the developments and trends in designing photonic structures for different photovoltaic applications.

## 1. Introduction

With increasing concerns over climate change, photovoltaics play a crucial role in the global transition to cleaner and more sustainable energy sources. Photovoltaics (PV) involve the conversion of light into usable electrical energy through photovoltaic cells, more often called solar cells (SC). Considering that the sun provides more than 10,000 times the current annual global energy consumption [1], researchers and engineers have focused on improving photovoltaic technology to make it more efficient, cost-effective, and widely applicable. The first practical solar cell was invented in 1954 by Bell Labs researchers [2]. It was made of silicon and had an efficiency of 6%. This breakthrough marked the beginning of photovoltaic technology, and currently, researchers are working on the fourth generation of solar cells [3]. Table 1 provides details of these generations.

**Table 1:** Various solar cell types with examples of obtained maximum efficiencies [3,4].

Generation	Type	Maximum Efficiency, %:	Literature about Using Photonic Crystals
First (Thick crystalline films)	Monocrystalline silicon	26.8 ± 0.4 [4]	
	Multicrystalline/polycrystalline silicon	23.3 [5]	
	III-V single junctions, e.g., GaAs thin film	29.1 ± 0.6 [4]	
Second (Thin films)	Thin film chalcogenide (e.g., CIGS)	23.35 ± 0.5 [4]	[6]
	Amorphous silicon (a-Si)	10.2 ± 0.3 [4]	[7]
	Microcrystalline silicon (mc-Si)	11.9 ± 0.3 [4]	[8]
Third (Emerging technologies)	Dye-sensitized (DSSC)	11.9 ± 0.4 [4]	[9]
	Quantum dots (QDSC)	18.1 [5]	[10]
	Organic (OSC)	15.7 ± 0.3 [4]	[11]
	Perovskite (PSC)	24.35 ± 0.5 [4]	[12]
	Multiple junctions, etc. (e.g., III–V 5J direct-bonded perovskite/Si cells)	38.8 ± 1.2 [4]	[13]
Fouth (Hybrid)	Metal nanoparticles and metal oxides (e.g., hybrid silver/silver oxide nanoparticles in OSCs)	5.2 [14]	[15]
	Carbon nanotubes (e.g., CNTs in OSCs)	14.37 [16]	[17]
	Graphene and its derivatives (e.g., MoS_2_ quantum dot/graphene with CH_3_NH_3_PbI_3_ perovskite)	20.12 [18]	[19]

According to the Shockley–Queisser limit, the maximum efficiency for a single-junction solar cell can reach approximately 33% [20]. The commonly achieved efficiency varies depending on the type of solar cell and still falls below this limit (see Table 1) (up to 26.7% [3,21]). It is possible to surpass this limit by using, for example, multiple-junction solar cells of the third generation, which can theoretically achieve an efficiency of up to 86.81% for an infinite number of monochromatic cells [22].

Solar cells are typically made using various materials, including silicon, cadmium telluride (CdTe), copper indium gallium selenide (CIGS), perovskites, and organic/polymers. The choice of material depends on factors like efficiency, cost, and the specific application. Silicon in various forms is still the most commonly used material [23,24] due to its natural abundance, environmentally friendly chemistry, high efficiency, and low cost [25,26]. Nonetheless, there are certain limitations associated with silicon solar cells. These include reduced efficiency caused by the indirect band gap of silicon, which results in the transmission of photons from the cell’s active area without generating charge carriers. Additionally, energy losses occur due to reflection on the top surface of the cell.

Generally, there are several factors connected with the interaction of light and material that contribute to the restrictions on the efficiency of solar cells:

- Bandgap Limitation: The bandgap of a material determines the PV conversion band, which means the range of wavelengths it can absorb. Also, the type of band, direct or indirect, affects the efficiency.
- Absorption limit connected to reflection and transmission losses: Not all sunlight that strikes a solar cell can be absorbed and converted into electricity. Some wavelengths of light may pass through or be reflected, reducing the overall efficiency. The ray optics theory states that the absorption in a bulk solar cell’s structure should not surpass the so-called Lambertian limit by conventional light-trapping [27].
- Recombination: Charge carriers (electrons and holes) generated by absorbed photons can recombine before reaching the electrodes, leading to losses in efficiency.
- Thermalization Losses: When photons with energy higher than the material’s bandgap hit the solar cell, their excess energy can be lost as heat instead of being converted into electricity. This effect simultaneously leads to decreasing efficiency due to increasing recombination rates and changes in material properties.

Researchers and engineers are continually working to address these limitations through innovative designs, materials, and manufacturing techniques to improve the efficiency of solar cells and make them more cost-effective. Minimizing the mentioned restrictions and enhancing solar cell output performance characteristics can be realized by applying various photonic structures [28], e.g., plasmonic nanostructures, nanocones, triangular and pyramid gratings, nanowires, and photonic crystals. Photonic crystals (PCs) are one of the best candidates for this purpose. They can selectively manipulate reflection and transmission spectra and enhance the interaction of light with material. Depending on the role, photonic crystals can be integrated with the structure of solar cells as an additional layer, or an active layer of SC can be designed as a PC structure. Table 1 includes information concerning exemplary applications of PCs in various solar cells.

## 2. Photonic Crystals

**Figure 1:**
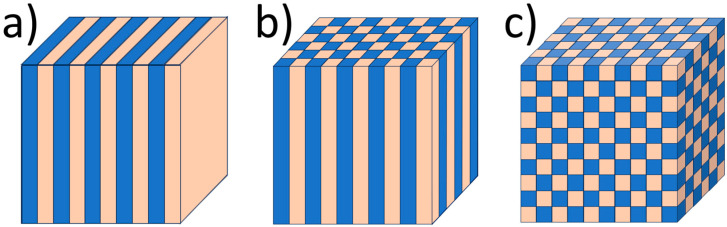
Models of photonic crystals: (**a**) one-dimensional (1D), (**b**) two-dimensional (2D), (**c**) three-dimensional (3D). The different colors represent materials with various refractive indices.

Photonic crystals are artificial structures with a spatial periodicity of dielectric permittivity on the wavelength scale. This gives rise to Bragg diffraction, facilitating both constructive and destructive interference effects for certain wavelengths of light, leading to a photonic band gap (PBG). Within this spectral region, light is unable to propagate through the material. The existence of the PBG profoundly impacts interactions between light and matter, enabling phenomena such as light confinement, waveguiding, trapping, enhanced nonlinear effects, increased light emission, and the slow photon effect [29]. Periodicity can vary from one-dimensional to three-dimensional with a period of the order of the wavelength of light, and according to this, PCs can be categorized into one-dimensional (1D), two-dimensional (2D), and three-dimensional (3D) photonic crystals (Figure 1).

**Figure 2:**
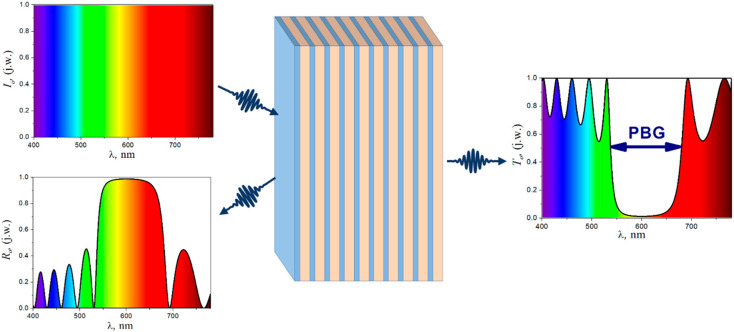
The Bragg mirror scheme and the reflectance and transmission spectra calculated for the Bragg mirror consist of 10 pairs of layers 75 nm thick and a refractive index of 2.0 and layers 100 nm thick and a refractive index of 1.5 [32].

The spatial period of structure “a” is called the lattice constant, analogous to the lattice constant in ordinary crystals, built of regularly arranged atoms [29]. The interaction of light with such periodic structures depends on their geometry, i.e., both on the type of crystal lattice, the shape of elements located in the nodes of the lattice [30], and the volumetric share of the material with a given refractive index in the unit cell, quantified by the value called the filling factor, f [31]. Another crucial parameter affecting such periodic structures’ properties is the contrast of individual areas’ refractive indices. The most crucial feature of photonic crystals is the photonic band gap (PBG) in the photonic band structure. Its presence can be verified by examining the spectral characteristics of the reflection or transmission (Figure 2).

### 2.1. 1D Photonic Crystals

1D PCs are otherwise called distributed Bragg reflectors (DBR) (Figure 2). Generally, they are designed as narrow-band reflectors. The maximum reflectance is centered at Bragg’s wavelength (λ*_B_*), referred to as the photonic band gap:

(1) $$m\lambda_{B}=2\left(n_{L}h_{L}+n_{H}h_{H}\right),$$

where *m* is the diffraction order and *h_L_* and *h_H_* are the thicknesses of layers with refractive indices *n_L_* and *n_H_*, respectively. In the 1D structure, the photonic band gap exists independent of the contrast in the refractive index. However, it influences the PBG range, Δω, and thus the range of high reflectance [33]:

(2) $$\Delta \omega =\frac{8c}{\lambda_{B}}arcsin\left(\frac{\left|n_{L}-n_{H}\right|}{n_{L}+n_{H}}\right)$$

### 2.2. 2D Photonic Crystals

**Figure 3:**
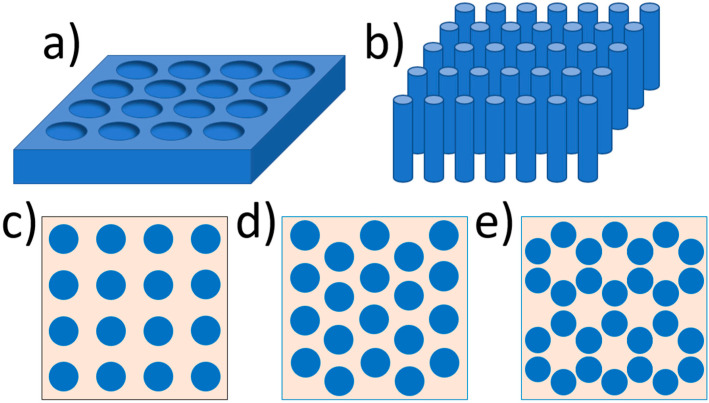
Exemplary 2D photonic crystals: (**a**) hole-type and (**b**) rod-type. Crystal lattices presented in 2D PCs: (**c**) square, (**d**) hexagonal, and (**e**) “honeycomb”.

Generally, two types of such crystals can be distinguished [34,35]: photonic crystals of the “hole” type (Figure 3a), consisting of cylinders with a low refractive index embedded in a medium with a high refractive index [36,37,38,39], and the “rod” type (Figure 3b), consisting of rods with a high refractive index surrounded by a medium with a low refractive index [35,40,41,42,43,44,45]. Both holes and rods can be arranged differently to create different 2D crystal lattices. A few examples are shown in Figure 3c–e.

The main factors determining the properties of two-dimensional photonic crystals are the refractive index contrast, the degree of filling with a material with a high or low refractive index, and the type of crystal lattice. In 2D structures, the formation of a two-dimensional PBG requires the fulfillment of additional conditions [46,47], such as the appropriate geometry of the structure and a sufficiently high contrast of refractive indices [47,48,49]. The greater the contrast, the wider the total PBG is.

Depending on the structure, the PBG may exist only for the TM or the TE mode [33,34,50]. Only in exceptional cases is a two-dimensional photonic band gap obtained for both polarizations [51,52], like in the “honeycomb” lattice.

### 2.3. 3D Photonic Crystals

**Figure 4:**
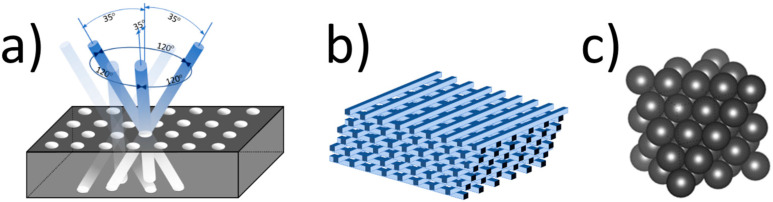
Examples of 3D PCs [33]: (**a**) Yablonovite, consisting of a triangular system of holes prepared by drilling the slab at a specific angle; (**b**) woodpile, formed “layer by layer” by a stock of dielectric 1D bars with alternating orthogonal orientations; (**c**) opal, obtained by self-organization from monodisperse colloidal suspensions.

A large variety of 3D PCs exist. Some of the most popular are presented in Figure 4.

Similar to 2D structures, the properties of 3D PCs depend on the geometry, degree of filling, and contrast in refractive indices [33].

**Figure 5:**
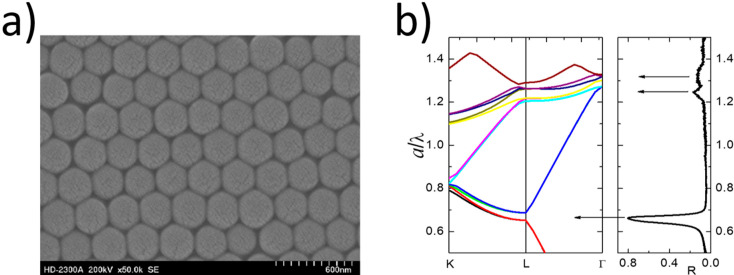
(**a**) Typical SEM micrograph of SiO_2_ bare opal [54] and (**b**) photonic bands calculated for SiO_2_ bare opal compared with measured reflectance spectra for a normal incidence of light on a (111) surface [53].

An exemplary 3D photonic crystal in the form of SiO_2_ opal and its corresponding photonic band structure compared with reflection spectra are presented in Figure 5 [53].

**Figure 6:**
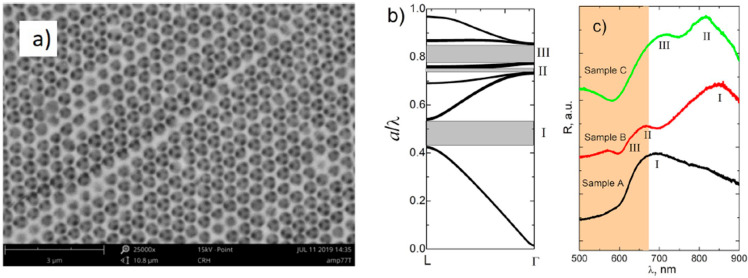
(**a**) Typical SEM micrograph of SbSI inverse opal; (**b**) photonic band structure of inverse opal calculated in the Γ-L direction with the PBG marked in grey; (**c**) reflection spectra of SbSI inverse opals registered in RT; spectra are vertically displaced for better clarity; the range of strongly absorbed wavelengths are marked with orange. Reprinted from [65], Copyright (2020), with permission from Elsevier.

It is much more challenging to produce three-dimensional structures with an appropriate, low periodicity (e.g., comparable to the wavelengths of visible light) than one- or two-dimensional structures. They require much more sophisticated and expensive technology like micromanipulation [55,56], wafer fusion bonding [57], interference lithography [58,59], X-ray lithography [60], holographic lithography [61,62,63], and ion beam lithography [64]. Producing the desired structure requires the use of a suitable method. Photonic crystals of the opal or inverse opal type are noteworthy. They can be formed due to the self-assembly of colloidal systems consisting of monodisperse spherical particles. It should be emphasized that this is the simplest and cheapest template production method that does not require expensive equipment. Simultaneously obtained layers have relatively good quality and a large surface. Using ordered spheres from several hundred nanometers to several micrometers results in a gap for electromagnetic waves in the visible and near-infrared range. Changing the sphere size shifts the wavelength range of the PBG. This fact influences the optical spectra registered for PCs. The authors of [65] presented reflection spectra in the range of 500–900 nm for three samples of SbSI inverse opal, which differed only with sphere size (see Figure 6). Additionally, they analyzed the influence of the absorption band edge (E_g_) of SbSI on such spectra.

The photonic crystal can redirect, concentrate, or even trap light incidents on it. Different materials (dielectrics, semiconductors, metals, polymers, etc.) and 1D, 2D, and 3D architectures (layers, inverse opal, woodpile, etc.) of photonic crystals enable great flexibility in designing the optical response of the material. This opens an extensive range of applications, among which are solar absorbers. This paper presents an overview of the latest developments and trends in applying photonic crystals for photovoltaics.

## 3. Photonic Crystals in Photovoltaics

Photonic crystals can be applied in solar cells to enhance their performance and efficiency through better light management and trapping. They can be used as:

1. Anti-reflective and light-trapping surfaces
2. Back reflectors
3. Spectrum splitters
4. Absorption enhancers
5. Additional light management layers
6. Radiation coolers (RCs)
7. Electron transport layers (ETLs)

Selected solutions are presented in subsequent chapters. Very often, the choice of structure is dictated by the technological capabilities required for PC production. However, one can also come across proposals for more sophisticated structures, such as photonic crystals comprising gyroidal and hyperbolic layers [66]. The advantage of such a structure is an angle-insensitive reflector for solar energy applications, such as reflectors, a wavelength-selective absorber, smart windows, and an intermediate layer for solar cells.

### 3.1. Anti-Reflective and Light-Trapping Surface

When electromagnetic radiation falls on the interface of two transparent media, generally, two phenomena occur: Part of the radiation passes from one medium to another, undergoing refraction, and the rest reflects from the interface. The more radiation is reflected, the less radiation passes inside. The bare silicon, still the most common material used in solar cells, in intimate contact with air, reflects approximately 35% of the incident power over all wavelengths used in solar cell work [67]. Thus, applying anti-reflective coating (ARC) or surface texturization is crucial to improve the efficiency of a solar cell [68,69]. They aid in preventing reflection and ensure as much radiation as possible penetrates the active layer, particularly of the wavelength range that interacts with the given material.

**Figure 7:**
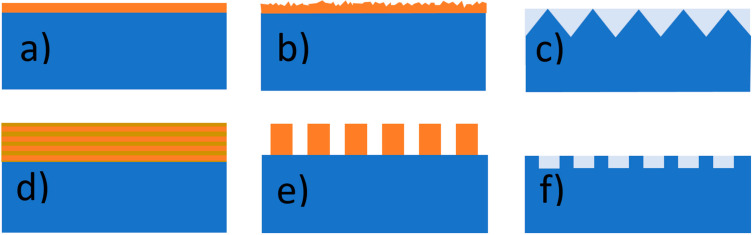
Different anti-reflective and light trapping surfaces: (**a**) interference thin film; (**b**) texturized thin film; (**c**) texturization in the form of a regular set of, e.g., micropyramids; (**d**) 1D photonic crystal; (**e**) rod-type 2D photonic crystal (**f**) hole-type 2D photonic crystal.

Different antireflection coatings have been optimized theoretically and experimentally so far (Figure 7). The simplest ARC consists of a thin layer of dielectric material (Figure 7a) with a specially chosen thickness so that interference effects in the coating cause the wave reflected from the ARC top surface to be out of phase with the wave reflected from the semiconductor surfaces [70]. The layer type and thickness depend on the material used in a solar cell. The dielectric contrast between the air and material of the solar cell should be reduced to reduce the reflection spectrum [71]. For instance, Fedawy et al. [70] examined the efficiency of GaAs solar cells covered with a Si_3_N_4_ antireflection layer. They increased efficiency from 14.89% to 27.16% by adding 75 nm Si_3_N_4_ ARC and 29.57% after additional front surface texturing (Figure 7b).

In the 1980s, Green et al. had already conducted a computer-based analysis of a double-layer antireflection phenomenon [68]. They found that a combination of a quarter wavelength of ZnS (refractive index, n = 2.25–2.35) and a quarter wavelength of MgF_2_ (n = 1.35–1.40) provided the best results (both thicknesses referred to a vacuum wavelength of 600 nm) in the case of silicon solar cells. It is possible to keep reflection loss below 2% from 460 to 940 nm with such a coating [68]. Multilayer antireflection structures (Figure 7d) have also been analyzed [72,73]. Womack et al. found that stacks of two SiO_2_ and two ZnO_2_ alternating layers of various thicknesses on thin film CdTe solar cells reduced their average reflection to ~1.2% [72]. Multilayer systems can be referred to as 1D PCs, provided that the periodicity of the layer system is maintained. These structures, commonly called distributed Bragg reflectors, are typically engineered to serve as narrow-band reflectors. However, their transmittance is almost negligible in the photonic band gap range wavelength. Regardless, applying 1D PCs as an ARC has been considered [26,69,71,74,75,76]. The consideration is often limited to the one-period photonic crystal structures [26,71,74,76], meaning multilayer structures are analyzed. The details of the examined 1D structure are presented in Table 2. Most of the works concern only theoretical considerations. An attempt to obtain the analyzed structure was made by Bennet’s group [75]. They used reactive sputtering to fabricate non-stoichiometric silicon oxynitride (Si_x_O_y_N_z_) layers whose refractive index ranges typically from 1.4 to 1.7, depending on content.

Generally, better results were achieved in the case of ternary 1D PCs (e.g., [71]). To enhance the absorption, the authors of [71] combined multilayer ARC with inverted pyramid texturing on the top layer.

Two-dimensional photonic crystals (see, for example, Figure 7e,f) can also be utilized for antireflection [77,78,79] and light trapping [80,81,82,83,84] covers (see Table 3). Two main types of optical effects are commonly used in such structures: one based on geometric optics and the other based on wave optics [85]. The first relates to the shape conducive to multireflection and consequently improves absorption. The second approach is based on the peculiar properties of photonic crystals.

In 2010, Hung et al. [77] applied 2D PCs with tapered rods to achieve antireflection and enhanced absorption. That structure reduces the reflection due to a gradually changed effective index. On the other hand, strong optical resonances for TM-mode can be found in this structure, mainly due to the complete photonic band gap inside the material. Such resonance can enhance the optical absorption inside the silicon PCs due to its increased optical paths [77]. In 2013, surfaces with micropyramids (Figure 7c) were used in commercial solar cells as part of an antireflection strategy [78]. Dominguez et al. [78] optimized 2D photonic crystals (PCs) onto Si wafers to improve the performance of c-Si solar cells. Their objective was to find a structure capable of minimizing the reflectance of the Si wafer in the spectral range between 400 nm and 1000 nm. They analyzed both hole-type and rod-type 2D PCs (see Figure 7e,f) with different shapes of holes (inverted pyramids, square holes, round holes) and rods (round pillars, cones, square pillars) and different crystal lattices (square, hexagonal, rectangular) [78]. The pattern of circular pillars arranged in a square lattice with a pitch of 448 nm, a diameter of 325 nm, and a height of 138 nm (a) was found to be the best structure, with an average reflectance of 3.6% in the spectral region from 400 nm to 1000 nm. This value was obtained without any other material used as an anti-reflective coating.

Shen Hong-Jun et al. [86] designed a solar cell with an antireflection layer in the form of regularly arranged dielectric cylinders with a truncated cone shape. They used Si_3_N_4_ to form cones and SiO_2_ as a substrate. [85]. Kuang et al. [81,87] proposed a unique teepee-like PC on crystalline silicon (c-Si). This structure is characterized by excellent antireflection due to its Gaussian-type gradient index profile. It also enhances light trapping due to its near-orthogonal energy flow and vortex-like field concentration via the parallel-to-interface refraction effect inside the structure. On the other hand, the structure optimized for light-trapping, e.g., a teepee-like structure, may act as a surface recombination center and reduce solar cell efficiency [84]. Bhattacharya and John state that the most likely candidate for high-efficiency silicon solar cells consists of inverted micropyramid PCs [21].

It is worth mentioning that some authors treat inverted pyramid structures as a type of texture [71,88], while others treat them as PCs [89,90,91], calling them surface PCs [91]. If the lattice spacing is comparable to the desired wavelength, light trapping should be enhanced by robust wave interference in the photonic structure [89] and resonant effects [91]. However, according to Razzaq et al. [92], the influence on the efficiency of random pyramid texturing is comparable with a periodic inverted nanopyramid structure. A further study [93] showed that an optimized inverse nanopyramid regular structure could outperform the random pyramid texture when considering incidence angle variations.

These structures, playing the role of ARC, simultaneously contribute to light trapping, absorption enhancement, radiation cooling, etc.

### 3.2. Back Reflector

It is vital to prevent light from escaping from the active layer without being absorbed. This condition requires a relatively thick active layer (even up to 3 mm) in most popular silicon solar cells [94]). Back reflectors are applied to reduce this thickness. They reflect the light passing through the active layer back to the active region and enhance the path along which light can be absorbed. The simplest back reflectors are metal layers [82,95], for example, Ag [81,94] or Al [94,96]. However, they have some drawbacks (plasmonic resonance loss of the interface between the metal and absorption layers, performance degeneration due to metal ion diffusion) [94]. PCs find their application in thin film solar cells by providing controllable and enhanced reflections from material layers to promote increased absorption. The wavelength range and intensity of reflection depend on the PBG and can be easily adjusted thanks to a geometrical dimension of the PC structure. It allows for the easy optimization of 1D PCs concerning specific requirements.

**Figure 8:**
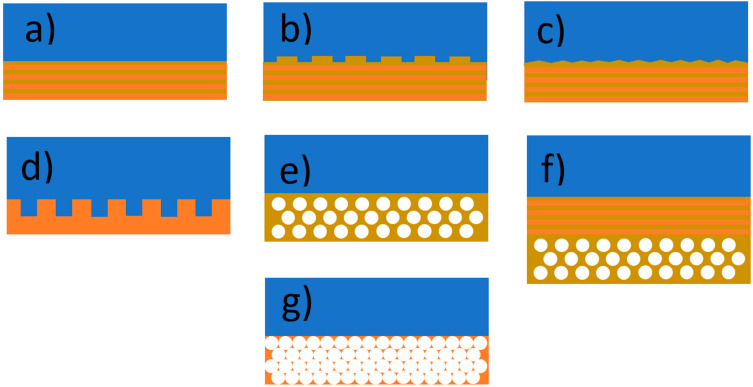
Different photonic crystal back reflectors: (**a**) 1D (DBR); (**b**) 1D with a reflection grating; (**c**) textured 1D; (**d**,**e**) 2D; (**f**) the combination of 1D and 2D; (**g**) 3D inverse opal.

There have been a large number of propositions for using PCs as a back reflectors (Figure 8):

- 1D binary PCs (DBR) [23,26,97,98,99,100,101,102,103,104]
- 1D ternary PCs [24]
- Combination of a reflection grating and 1D PCs [105,106]
- Textured conductive PCs (TCPC) [107]
- 2D PCs [108,109]
- Combination of 1D and 2D PCs [94]
- 3D inverse opals [110,111]

**Figure 9:**
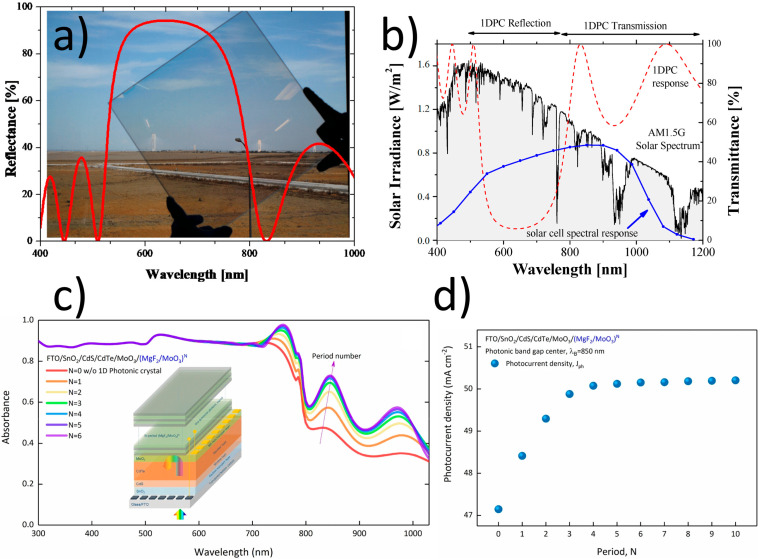
(**a**) A 1D PC designed to reflect in the visible solar spectrum with the corresponding reflection spectrum; (**b**) comparison of the visible solar spectrum, PC transmittance (dashed red line), and silicon solar cell spectral response (blue dots) [99]; (**c**) the scheme of SCs containing 1D PCs and their absorbance spectra calculated for different periods at λ_B_ = 850 nm [103]; and (**d**) variation in J_ph_ according to the number of periods [103]. Figure 9a,b are reproduced from [99], licensed under a Creative Commons Attribution (CC BY) license, Figure 9c,d are reproduced from [103], licensed under a Creative Commons Attribution (CC BY) license.

Thanks to the PBG, photonic crystals can reflect up to 100% [95,102] of incident light over the PBG wavelength range. That is challenging to achieve using metal and dielectric mirrors. One condition is that this phenomenon refers only to the wavelengths in the range of the PBG. The key parameter for optimizing the PC design consists of adjusting the reflection peak (Bragg peak) to the solar photon energy to reflect it. This structure should reflect light within the range where the active material responds most efficiently. Figure 9a shows the PC comprised of SiO_2_ and TiO_2_ layers deposited on glass by the DC magnetron sputtering technique by Delgado-Sanches and Lillo-Bravo [99] and the corresponding reflection spectrum. In this example, the device reflects photons within the visible range while the near-infrared spectrum is transmitted, as shown in Figure 9b. They observed that solar irradiance harvesting enhancement occurred when the sun’s elevation angle was between 50° and normal incidence [99].

Çetinkaya et al. [103] examined the structure of SCs composed of FTO/SnO_2_/CdS/CdTe/MoO_3_/(MgF_2_/MoO_3_)^N^ layers. They presented the influence of a number of periods on the absorbance of light (Figure 9c) as well as on photocurrent density (J_ph_) (Figure 9d). They noticed an increase in absorption and photocurrent density after implementing the PCs. At the same time, the periodicity of the PCs can be restricted to four periods due to saturation effects. Doghmosh et al. [24] studied ternary 1D PCs theoretically. By introducing a third 30 nm layer of Si_3_N_4_ between every two layers of SiO_2_ and Si, the omnidirectional region was improved by approximately 16%.

Zeng et al. [105] and Zhou et al. [106] analyzed the combination of 1D PCs as a DBR and conventional [105] or 2D PC [106] reflection grating. With such a combination, the authors of [106] efficiently harvested solar photons without losses associated with textured metallic reflectors. They demonstrated that for near band edge photons, the optimized solar cells reach more than a hundred-fold increase in path length at diffraction resonances and exceed the classical light path enhancement limit predicted for randomly roughened interfaces.

Chen et al. [107] proposed another light trapping scheme based on a textured conductive photonic crystal (TCPC) back reflector in an n-i-p hydrogenated amorphous silicon (a-Si:H) solar cell. TCPCs combine a flat 1D PC and a randomly textured layer of chemically etched ZnO:Al. Total efficiency enhancement was obtained thanks to the sufficient conductivity, high reflectivity, and intense light scattering of the TCPC back reflector.

The other proposition of using a 2D PC as a back reflector for CdTe solar cells is presented in [109]. The proposed 2D structure has a hexagonal lattice of Ge rods placed in the SiO_2_ background. A device supplemented with such a structure has approximately 5% more power conversion efficiency than solar cells with metal back reflectors.

Zhan and Cai [94] obtained a back reflector in a full-wave band of 470–1100 nm by combining 1D and 2D PCs. The applied 1D PC was SiO_2_/c-Si DBR, and the 2D PC was a crystalline silicon slab with etched periodic air pores. The reflectivity of it is calculated to be 97.85%. Considering only the 2D PC and a range of 800–1100 nm, they achieved 99.32% reflectivity.

Varghese et al. [110] experimentally demonstrated a-Si inverse opal PCs as back reflectors for Si solar cells. The authors observed increased absorption of near-IR wavelengths and a 10% enhancement in the short-circuit current with no degradation in the open-circuit voltage. Additionally, they proposed simplifying the PC integration with a solar cell by transferring the free-standing PC membrane to form the PC onto electrically contacted cells. Simulations performed by the authors of [111] on such c-Si solar cells with inverse opal indicated the best effects were obtained for spheres with a diameter of 1 μm. The power conversion efficiency reached 30.4% compared with 20.95% without a back reflector.

### 3.3. Spectrum Splitter

**Figure 10:**
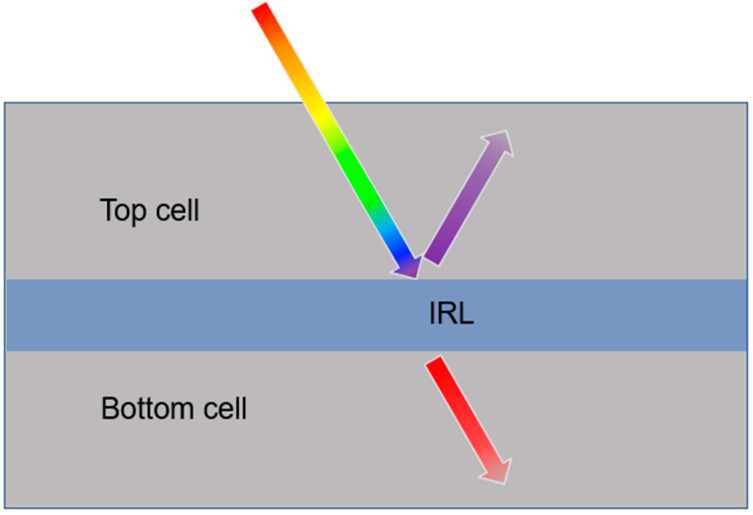
Schematic light propagation through a tandem solar cell structure with IRL.

Tandem solar cells are created for conversion power over a wide energy spectrum. Such cells consist of two or more layers of material having different energy gaps. The layer with the higher energy gap is placed at the top, and the layer with the lower energy gap is placed at the bottom (Figure 10). The most commonly used combination of materials is c-Si and a-Si [112]. Such a structure requires an additional intermediate reflected layer (IRL) for spectrum splitting and light management between these layers. The non-absorbed photons with shorter wavelengths should be reflected to the top layer of the solar cell, and the photons with longer wavelengths should be transmitted to the bottom one [69]. Photonic crystals are perfect for designing an IRL (see Figure 2). Moreover, the IRL must possess adequate electrical conductivity to prevent any losses through ohmic resistance in the interconnection of the active layers [25].

1D multilayer structures [69,113], 1D photonic ribbon structures [114], and 3D inverse opals [25,113,115] as IRLs have been considered theoretically and experimentally. O’Brien et al. [113] compared solar cells’ properties with different IRLs, like 1.5-bilayer and 3.5-bilayer μc-Si/ZnO (acting as Bragg reflectors) and ZnO inverse opal. One of their conclusions was that the Bragg reflectors provide a larger band gap and less parasitic absorption than the analyzed inverted opal PC. The constituent materials of 1D binary PCs designed for c-Si/a-Si tandem solar by Sayed et al. [69] were Bi_4_Ge_3_O_12_ and μc-SiO_x_:H. They examined ten-period structures consisting of a 62 nm layer of Bi_4_Ge_3_O_12_ and μc-SiO_x_:H layers with different thicknesses (40 nm, 55 nm, and 73 nm) depending on the sample. However, they observed the widest PBG (400–730 nm) when stacked with all three structures.

Sundar et al. [112] theoretically designed a 1D magnetic photonic crystal (MPC) as a multilayer system of a metal-doped magnetic composite material (Cu-YIG) and μc-SiO_x_:H. They found that the photonic bandgap of MPC increases with the number of periods and the percentage of copper doping, which tends to enhance the light trapping in tandem solar cells.

A completely different 1D structure was demonstrated by Amiri et al. [114]. It was a one-dimensional silicon strip with a 200 nm breadth and a length of 18 μm with ten air holes etched on it. The air hole diameter was 180 nm and the lattice spacing was 1.75 μm. The structure parameters were matched to reflect the light of wavelengths from the specific range thanks to a photonic band gap. The reflected signal was absorbed by the upper cell, and the transmitted signal was absorbed by the bottom cell.

The other type of investigated IRL is inverse opal [25,113,115]. In 2008, Bielawny et al. [25] prepared a thin film of ZnO inverse opal at the rear side of a-Si solar cells with the intention of later integration with c-Si/a-Si tandem solar cells. Upping et al. [115] examined the impact of the IRL in the form of ZnO:Al inverse opal on the c-Si/a-Si tandem solar cell external quantum efficiency. They reported an increase in the enhancement of the external quantum efficiency in the a-Si:H layer with a factor of 3.6.

### 3.4. Absorption Enhancer

The Lambertian limit serves as a crucial benchmark in assessing the efficiency of solar cells [116]. Research has indicated that both extremely thick and extremely thin solar cells can approach this limit by implementing effective photon management strategies. By optimizing techniques for managing photons, these cells can operate closer to the Lambertian limit, resulting in improved overall performance. One way to enhance absorption is to use an active layer of SC in the form of a PC. Ordered photonic crystals have highly localized diffraction profiles and offer strong coupling to optical modes at specific wavelengths, with corresponding absorption enhancements that can exceed the Lambertian limit [117]. A periodic structure leads to nonlinear dispersion, which results in flat photonic bands and the appearance of band gaps in photonic crystals. The group velocity of light with wavelengths close to band gaps is anomalously low, and the so-called slow photon effect is observed [118]. The lower light velocity increases the effective optical path of light [119]. Thus, when the edge of the PBG overlaps with the electron absorption edge, the result can be expected to enhance light absorption [119]. The absorption edge can be tuned by varying the dimensions of the periodic structure, thereby moving the PBG [120]. This effect occurs for the photonic band gap’s red and blue edge [121]. Many authors explain absorption enhancement by the existence of slow photons [11,121,122,123,124]. However, Mihi and Miguez [121], examining dye-sensitized SCs as a combination of a photonic crystal and a layer of nanocrystalline absorbing material, concluded that absorption enhancement due to resonant modes localized within the absorbing coating brings a better effect than slow photons in the active layer of the PC form. Therefore, photonic crystals play only the role of porous colloidal mirrors operating through coherent scattering [121,125]. Coupling to the resonant modes was also considered by other authors [91,116,126,127], even in the case of the PC active layer [127]. Another explanation based on tuning the coupling strength of incident radiation to quasi-guided modes over a broad spectral range using the PC structure appears in refs. [128,129,130,131]. Such coupling enables the photons to spend enough time in the patterned active layer to be absorbed there [127]. The other mechanism for absorption enhancement in photonic crystals arises from strong resonances from parallel interface refraction (PIR) [132]. This anomalous refraction type is negative and usually out of the plane of incidence. Light impinging on photonic crystals over a wide range of frequencies couples to Bloch modes and propagates nearly parallel to the thin film-to-air interface. This phenomenon leads to anomalously long optical path lengths and a long time before the light beam exits the thin film. This effect can be much stronger than that of slow photons.

**Figure 11:**
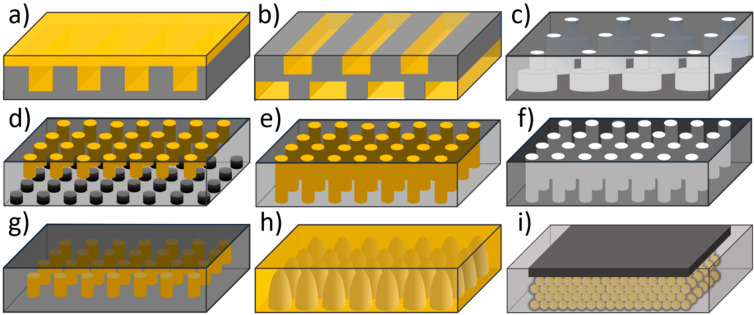
Examples of PC structures in the active layer: (**a**) 1D grating based on [129]; (**b**) double 1D grating based on [11]; (**c**) double layer of 2D PCs based on [128]; (**d**) double 2D PCs based on [116]; (**e**) 2D lattice of cylinders based on [123]; (**f**) 2D lattice of air cylinders based on [7,127]; (**g**) 2D PCs based on [124]; (**h**) lattice of nanocones based on [131]; (**i**) inverse opal based on [125].

Various photonic crystal structures, for example, 1D grating [11,129,133], 2D structures [7,116,123,124,127,128,130,131,134,135], and 3D structures [121,122,125,136] have been investigated for solar cell applications (see Figure 11).

#### 3.4.1. 1D Photonic Crystal

1D PCs were theoretically and experimentally considered by Duche et al. [11] to enhance absorption in the active layer in organic solar cells. Their structure consisted of two layers with 1D gratings (Figure 11b). One of them was the active layer made of a blend of PCBM ([6,6]-phenyl-C61-butyric acid methyl ester) and one of the polymers: P3HT (poly-3-hexylthiophe’ne) with an energy band gap E_g_~1.88 eV or TDPTD (poly(3-(2-methyl-2-hexyl-carboxylate) thiophene-co-thiophene)) with E_g_~1.83 eV. Due to slow photons, they demonstrated photonic absorption gains for the integrated spectra on an 8° incident cone, 3.36% and 15.67% for P3HT:PCBM and TDPTD:PCBM, respectively [11]. Merabti et al. [133] theoretically explored the feasibility of 1D PC grating in the active layer of an a-Si:H-based photovoltaic cell. This structure led to more significant electro-optical gains thanks to the interference effects and coupling of incident light with slow Bloch modes.

#### 3.4.2. 2D Photonic Crystal

2D structures have been the most often chosen PCs in designing solar cells for absorption enhancement.

Mallick et al. [128] restructured the active layer in ultrathin c-Si solar cells by patterning a 400 nm-thick c-Si layer into a double-layer PC with holes (Figure 11c). The holes in the upper layer had a smaller radius than those in the lower layer. This results in an enhancement of the maximum achievable photocurrent density from 7.1 mA/cm^2^ for an unstructured film to 21.8 mA/cm^2^ for a structured one, approaching the Yablonovitch light-trapping limit of 26.5 mA/cm^2^ for the same volume of active material. De Zoysa et al. [116,126] discussed enhancing broadband light absorption in the wavelength range of 600–1000 nm by utilizing multiple large-area resonant modes at the band edge of a PC in the case of ultrathin μc-Si film (Figure 11d). They achieved a high active-area current density of 22.6 mA cm^−2^ and obtained an active-area efficiency of >9.1% using a square-lattice 2D PC. Gomard’s team [7] studied 2D PCs for absorption enhancement in a-Si:H thin film. Their 100 nm-thick active material, patterned with a lattice of holes with squared symmetry, reached a high absorption of the incident light despite thickness below the diffusion length of the minority carriers [7]. Another proposal for improved light trapping in Si was presented in [135]. The approach was based on the quasi-resonant absorption of photons in a tandem arrangement of partially disordered photonic crystal plates separated by a nanoscale gap. This construction made it possible to surpass the Lambertian limit.

2D PC structures have also been implemented in organic solar cells [124,129,130,137,138]. Tumbleston et al. [129,130,138] attempted to enhance absorption in the active layer of organic solar cells by designing them in a photonic crystal structure: square posts with 395 nm 2D square periodicity and channels with 400 nm 1D periodicity. The research of Tumbleston’s team [129,137,138] on photonic crystal photoactive layers resulted in an increase in absorption by ~17% over the entire spectral range due to band edge excitation of quasi-guided modes [130]. The proposed active layer consisted of the poly-3-hexylthiophene/[6,6]-phenyl-C61-butyric acid methyl ester (P3HT:PCBM) bulk heterojunction blend and a porous form of low index of refraction (~1.4) conducting nanocrystalline zinc oxide (nc-ZnO). Additionally, PC geometry created excitons closer to the P3HT:PCBM exit interfaces [129]. Thus, free carriers might be more suited to escape from the photoactive blend, enhancing electrical performance readily. Another way of implementing 2D PCs in organic SCs was presented in [124] (Figure 11g). The author theoretically designed semitransparent organic solar cells with 2D photonic crystals inside the active layer, which was composed of two fullerene materials. She also concluded that PTB7-Th:PC71BM could be a better choice as an active layer than the P3HT:PCBM [124].

The improvement of absorption due to coupling incident light into quasi-guided modes has been investigated by Dottermusch et al. [131] in CuInSe (CIS) nanocrystalline-based solar cells. The 2D photonic structure was in the form of photoresist polymer nanocones of half-ellipsoidal shape arranged in a square lattice and embedded into a layer of CIS nanocrystals (Figure 11h). It resulted in absorption enhancement of only 3–7% because the low refractive index of the CIS nanocrystals was the main limiting factor that led to a restricted number of quasi-guided modes [131].

Another group of solar cells where an active layer was designed in the 2D PC were perovskite SCs [123,127,139]. The PC structure examined in [127] comprised air holes arranged into a hexagonal lattice (Figure 11f) in perovskite MAPbI_3_. The absorption enhancement was approximately 44% better than planar material [127]. A tetragonal lattice of indium arsenide (InAs) cylinders in the absorption layer of methylamine lead iodide (CH_3_NH_3_PbI_3_, MAPbI_3_) (Figure 11e) was presented in [123]. Implementing a photonic structure increased absorption efficiency up to 82.5% in the wide wavelength range of 400–1200 nm. This is much higher than the absorption layer without the PC structure. In addition, the absorption layer with photonic crystal presented a stable absorption efficiency of 80% in the wide incident range of 0–80°.

#### 3.4.3. 3D Photonic Crystal

The only 3D structure of PC considered so far for the active layer of solar cells is inverse opal [121,122,125,136] (Figure 11i). It has been used in dye- and quantum dots-sensitized solar cells. Already in 2003, Nishimura et al. [118] noticed a ~26% increase in the short circuit photocurrent efficiency across the visible spectrum (400–750 nm) by coupling a photonic crystal to a dye-sensitized nanocrystalline TiO_2_ photoelectrode.

Mihi and Miguez [121] observed absorption enhancement in various spectral regions depending on the size of the spheres and, thus, depending on the position of the PBG relative to the absorption edge while studying dye-sensitized nc-TiO_2_ inverse opal. This effect occurs for the photonic band gap’s red and blue edges. However, they concluded that better outcomes were achieved in the structure consisting of PCs and a bulk layer of absorbing material than in the photonic crystals only. The absorption enhancement occurs in resonant modes localized within the absorbing coating rather than in the photonic crystals. The latter plays the role of a mirror operating through coherent scattering. This was confirmed experimentally by Lee et al. [125]. Bayram and Halaoui [122] examined solar cells based on quantum-confined CdSe-sensitized TiO_2_ photonic crystals to amplify solar energy conversion. They observed an almost sevenfold amplification of the photon-to-current conversion efficiency for the examined inverse opal structure compared to a nanocrystalline TiO_2_ with similar CdSe sensitization [122]. The authors attributed this enhancement to a blue-edge slow photon effect due to overlapping the photonic band gap at 700 nm with the CdSe absorption edge (600–650 nm). The quantum dot Q-CdTe/Se layer-sensitized TiO_2_ inverse opal featured a four-fold enhancement factor of the photon-to-current conversion [136].

### 3.5. Additional Light Management Layer

Photonic crystals have also been introduced into SCs as light management layers [140,141,142]. As mentioned in the previous section, in dye-sensitized solar cells, better effects were achieved when colloidal PCs were used as an additional layer rather than the active one alone [121]. In [143], an additional multilayer colloidal structure composed of spheres with different diameters led to light harvesting enhancement of approximately 60% compared to standard dye-sensitized solar cells due to the mirror behavior of the colloidal superlattice.

**Figure 12:**
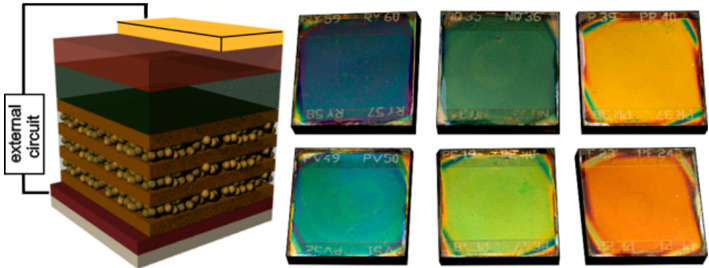
The PC is made by alternating deposition of SiO_2_ nanoparticles and TiO_2_ layers [140]. Reproduced from [140], licensed under an ACS Author Choice License.

Zhang et al. [140] integrated photonic crystals in the form of multilayer structures composed of dense TiO_2_ and porous SiO_2_ with perovskite solar cells (Figure 12). The resulting SC, in addition to high efficiency due to well-defined reflectance bands, can be aesthetically attractive as a result of its tunable structural color over the entire visible spectrum, depending on the thickness of the individual layers.

Buencuerpo et al. [141,144] simulated an ultrathin GaAs solar cell including additional PC layers: only on the front side, only on the back side, and on both the front and back sides of the active layer. The front layer was composed of TiO_2_ cylinders arranged in a square lattice inside an ARC of MgF_2_ when used alone or arranged in two displaced square lattices combined with the back PC layer. The latter was made of AlGaAs cylinders arranged in a square lattice inside a SiO_2_ spacer.

Producing periodic nanostructures is challenging and expensive; thus, the proposition of less demanding quasi-random photonic structures (e.g., disordered structures with a precisely defined reciprocal space distribution of spatial frequencies) has appeared [142,145]. An additional layer built into the back of GaAs solar cells led to a 10% relative improvement [142].

### 3.6. Radiation Cooler (RC)

An inherent effect of the work of solar cells is their heating. This undesirable effect adversely affects the efficiency of solar-to-electricity conversion and the lifetime of photovoltaic panels. The efficiencies of different PV technologies decrease with temperature, e.g., the efficiency of crystalline Si modules decreases at a rate of 0.45%/°C [146,147]. In the long run, high temperature activates and accelerates such unfavorable processes as contact corrosion and polymer degradation. As a result, in hot climates, the power degradation of crystalline Si photovoltaic modules progresses at approximately 1.8% per year, which is almost nine times faster than modules installed in cold climates (approximately 0.2% per year) [148]. In hot climates, the module lifetime was less than 15 years, well below the standard 25-year warranty for solar panels. Lowering the temperature of the cells is, therefore, a key factor in improving their efficiency and lifetime, which is vital in producing electronic waste. It is critically important to develop effective, practical, and preferably passive cooling methods to reduce the operating temperature of photovoltaic (PV) modules. This can be achieved by using the radiative cooling effect (RC), i.e., the process of heat loss by thermal radiation that can pass through the atmosphere into outer space. It is possible in the so-called atmospheric transparency window, i.e., in the 8–13 µm wavelength range. Materials that can absorb energy and radiate it in those wavelengths exhibit a strong cooling effect. However, it must be considered that for photovoltaic applications, lowering the operating temperature by RC is only viable if sunlight absorption can be maintained simultaneously.

Commercial solar cells are usually encapsulated with polymer or glass covers [149] and have a glass cover on top [150]. This glass protects delicate elements of PV modules from dirt, moisture, and mechanical damage. This glass is also a cooling element due to its inherent RC capability. Even though glass is already highly emissive in the IR region, it is still imperfect. Therefore, efforts are underway to produce materials that could support or replace glass in heat dissipation and be used to cool photovoltaic panels. Nanostructured materials, especially photonic crystals, may play an essential role. The thermal emission of photonic crystals was noticed and studied very early. Enhancement and suppression of thermal emission of radiation have been theoretically and experimentally demonstrated, among others, in 1D [151,152,153,154], 2D [155,156,157,158,159], and 3D [160,161,162,163,164,165,166] photonic crystals, as well as in structures composed of 2D and 1D photonic crystals [167]. However, not all of them met the requirements for solar cell radiative coolers, e.g., because of the significant reflection of incident solar irradiance.

Gao et al. [168] argue that “an optimised radiative cooler should exhibit the following characteristics: (1) its substrate should not be constrained (i.e., the cooler can be manufactured to be rigid or flexible), (2) it should be capable of large-area manufacture, (3) have photonic crystal structures with wide emissive angles, (4) be inexpensive, (5) have a near-ideal emittance profile, and (6) be capable of reducing the operating temperature of solar cells. However, manufacturing a radiative cooler with the above characteristics is still challenging”.

**Figure 13:**
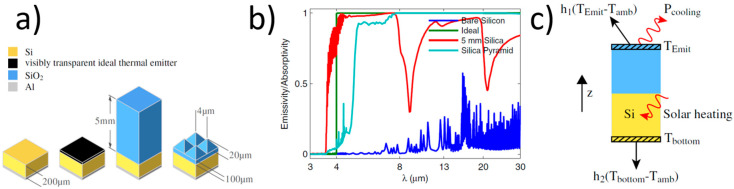
(**a**) Crystalline silicon solar cell structures with no thermal emitter, an ideal thermal emitter, a uniform silica layer, and a uniform silica layer with 2D silica pyramids [159]); (**b**) a comparison of emissivity/absorptivity spectra [159]; and (**c**) the thermal scheme assumed by ref. [159]. Reproduced from [159], licensed under the Open Access Publishing Agreement.

Zhu et al. [159] conducted a theoretical analysis of the cooling properties of a 2D square lattice of silica pyramids with 4 μm periodicity and 20 μm height on a 100 μm-thick uniform silica layer placed on a typical solar panel with no thermal emitter, an ideal thermal emitter, and a uniform silica layer (see Figure 13a).

The silica pyramid structure has an emissivity very close to unity, with no dips over a wide range of mid-infrared wavelengths (Figure 13b). The broadband absorption close to the ideal is because the pyramids provide a gradual refractive index change to overcome the impedance mismatch between silica and air at a broad range of wavelengths, including the phonon–polariton resonant wavelengths. Zhu et al. [159], assuming the thermal scheme presented in Figure 13c, found that the silica pyramid design substantially lowers the temperature of the solar cell. At 800 W/m^2^ solar irradiation, the temperature reduction in the silica pyramid design is 17.6 K compared with the bare solar cell. Furthermore, the silica pyramid is also optically transparent and does not reduce the absorption of solar energy; therefore, it is a promising cooling structure for photovoltaics. Zhu et al. [169] practically tested the efficiency of radiation cooling of photovoltaic panels using a 2D photonic crystal in the form of air rods in a silica matrix produced via photolithography. They also obtained a significant increase in emissivity to a near-unity value over the entire thermal wavelength range. Tests in rooftop conditions showed an, on average, 5.2 °C lower structure temperature with the silica photonic crystal than the bare absorber structure and over 1.3 °C lower than the absorber structure with the planar silica layer.

Zhao et al. [170] proposed and analyzed a photonic structure composed of 1D and 2D photonic crystals that selectively reflect solar radiation and actively radiate heat to outer space while maintaining its solar transmission in the PV conversion band (0.3–1.1 μm). The 1D photonic crystal forms a multilayer stack of alternating SiO_2_ and TiO_2_ layers (30 layers in total) deposited on a single layer of MgF_2_. On top of this stack is placed a 2D photonic crystal which, like in [169], is a square lattice (period = 6 μm) of air rods (diameter = 5 μm, depth = 10 μm) etched into a uniform layer of silica (thickness = 500 μm).

The structure designed in this way is characterized by close-to-unity transmission and, thus, the low reflection of sunlight in the range of 0.3–1.1 μm. However, in the entire range of mid-infrared waves and the atmospheric transmission window, the emissivity/absorptivity of this structure is very high. Additionally, the structure is a good reflector for near-infrared radiation above 1.1 μm (see Figure 4 in [170]). In their thermal simulation, Zhao et al., like Zhu et al. in [159], also considered other nonradiative heat dissipation mechanisms. The proposed photonic structure on top of the solar cell lowers its operating temperature by 10 °C. It is 7 °C lower than achieved for SCs with a glass coating on top and only 1 °C higher than SCs with an ideal heat emitter.

Gao et al. [168] made and experimentally tested the effectiveness of a photonic structure formed by a square lattice of 2D truncated cones deposited on a polyethylene terephthalate (PET) substrate. They designed the optimal structure using numerical simulations for various shapes, sizes, and periodicity to maximize emittance in the atmospheric transparency window. For the fabrication, Gao et al. used the UV nanoimprint method on a UV-curable adhesive (UVA) [168], which is much more economical than, for example, photolithography. The rooftop measurement showed that the photonic structure reduced the temperature of the underlying silicon wafer by approximately 1.2 °C. A much better effect was achieved when an additional layer of Ag was applied to the PET substrate. This layer significantly reflected solar radiation, which helped reduce the temperature by 7.7 °C but was not beneficial for the efficiency of solar cells.

A similar 2D structure in the form of SiO_2_ pillars was examined by Long et al. in [171]. They theoretically concluded that in extreme cases, for no convective heat coefficient (no wind), the designed structure should reduce the temperature of the solar cell by 16 °C. Simultaneously, they obtained a reduction of 2 °C compared with the bare structure during the rooftop measurement.

Unlike previous structures, Silva-Oelker and Jaramillo-Fernandez [172] numerically analyzed 2D structures based on hemispheres and a flat surface placed on a silicon photovoltaic cell. Considering different geometrical parameters, they determined maximum power improvements of 18.1% and 19.7% when using soda-lime and polydimethylsiloxane (PDMS) hemispheres, respectively, and a temperature reduction of 4 °C compared to a glass encapsulated solar cell.

The radiative cooler proposed by An et al. [173] integrates a multilayer thin-film stack and a SiO_2_ grating. They performed a comparable theoretical study on the performance parameters of the SCs with and without the radiative cooler. In this way, they showed that the SC temperature can be reduced by over 10 °C and the absolute power conversion efficiency (PCE) can be increased by 0.45% by employing the photonic radiative cooler. Zhao et al. [174] performed an outdoor experiment with silica micrograting as a cooler. Their structure fabricated through the etching process has a periodicity of 7 μm, a duty ratio of 0.2, and a vertical depth of 10 μm. The grating was highly transparent to sunlight and reduced the temperature of the commercial silicon cell by over 3.6 °C after adding it on top. Additionally, the proposed silica grating reinforced the light-trapping effect of the solar cell.

The 3D photonic crystal with an opal structure also has good cooling properties. [175]. It should be emphasized that opals are easy to produce and cheap. Besides cooling effects, applying opals with different sphere sizes caused the desired colorization. Kim et al. [175] implemented self-assembled silica opals on a silicon (Si) wafer. Daytime radiative cooling of the c-Si by as much as 13 °C while maintaining the nonabsorbing colorization is achieved through colloidal suspension coating.

**Figure 14:**
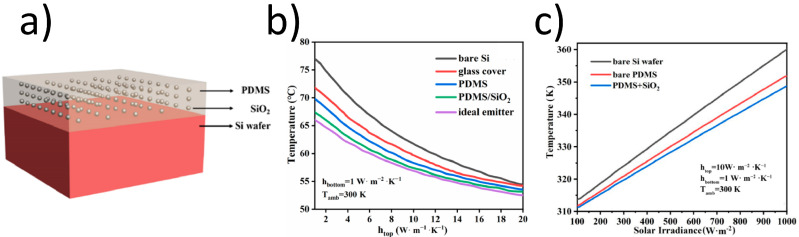
(**a**) Diagram of a PDMS/SiO_2_ radiative cooling system [176]; (**b**) comparison of operating temperature as a function of heat transfer coefficients and (**c**) solar irradiance for different radiative cooling systems [176]. Reproduced from [176], licensed under Optica Open Access Publishing Agreement.

Tu et al. [176] designed a difunctional coating for both RC and ARC for silicon solar cells. This coating is a PDMS layer with regularly arranged SiO_2_ particles (Figure 14). The best effect was theoretically obtained for the layer with a thickness of 55 μm, filling in 8% of the volume with SiO_2_ spheres of a radius of 500 nm. This PDMS/SiO_2_ radiative cooler can significantly lower the temperatures of c-Si solar cells by 9.5 °C, avoiding a 4.28% efficiency loss. Under the same conditions, the standard glass cover reduces the cell temperature by only 5.1 °C.

### 3.7. Electron Transport Layer

ETLs are conductive scaffolds coated with perovskite, which extract electrons from perovskite and deliver them onto a transparent electrode, blocking charge recombination [177,178] and eliminating the space charge [179]. Mesoporous TiO_2_ [178], ZnO [138,179], and PFN [85,179] are the materials applied in ETLs. The electron transport layer’s microstructure plays an important role that invariably affects the perovskite infiltration, light trapping, harvesting, and charge injection, transportation, and collection at the ETL/perovskite interfaces. Highly oriented arrays of nanorods, nanowires, and nanotubes have been widely used as ETLs in PSCs [180,181,182].

**Figure 15:**
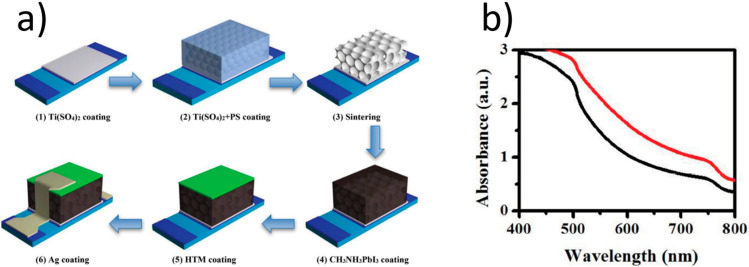
(**a**) Fabrication procedure of the inverse opal-like TiO_2_ electron transport layer-based perovskite solar cells. [183]; (**b**) absorption spectrum of the perovskite-coated TiO_2_ inverse opal ETL film (red line) and conventional P25 mesoporous film (black line) on the FTO glass [183]. Reproduced from [183], licensed under a Creative Commons Attribution (CC BY 4.0) license.

Chen and co-workers [183] introduced TiO_2_ inverse opal as an ETL for the first time in 2015. It was prepared using a simple polystyrene assistant method (Figure 15a). The layers combined the functions of the compact and mesoporous scaffold layers in perovskite solar cells. The porous structure of inverse opal could enhance the devices’ light harvesting efficiency. It maintained excellent transmittance of short wavelength light. Also, it promoted the transmittance of long wavelength light, while the conventional P25 mesoporous film demonstrates lower transmittance than bare FTO in the wavelength region from 300 to 600 nm. Due to the additional antireflection property, more light will arrive at the perovskite layer supported by the proposed ETL. As a result, better absorbance (Figure 15b) and power conversion efficiency (13.11%) were obtained in comparison with the conventional P25 mesoporous layer (11.00%). Moreover, the bottom of inverse opal ETLs plays the same role as the traditional compact layer, transporting electrons and inhibiting the recombination of electrons and holes.

Kim et al. [184] examined a layer with a TiO_2_ hemisphere structure which was prepared using the nanoimprint technique. By controlling the dimension of the hemispheres, they concluded that the 1400 nm-sized hemisphere pattern using 1600 nm polystyrene beads provided the highest light-utilization efficiency among those in the visible range. In addition, the recombination rate of the electron transport layer was also decreased. As a result, the power conversion efficiency of perovskite solar cells was improved from 10.5 to 15.2%.

Another ETL monolayer structure was reported in [185]. It was a well-organized monolayer SnO_2_ inverse opal. Its periodic structure exhibited an optical coupling phenomenon, enhancing the perovskite layer’s light absorption. Furthermore, the well-organized structure with appropriate pore size triggered the confined crystallization of perovskite films and optimized the interface of SnO_2_/perovskites, suppressing the interfacial electron–hole recombination. As a result, the power conversion efficiency of mesoporous perovskite solar cells fabricated was boosted from 19.63% for the control device with a layer of mesoporous SnO_2_ to 22.01% for devices with a SnO_2_ inverse opal layer as an ETL.

Zheng et al. [186] presented a dual porous TiO_2_ ETL for carbon cathode-based perovskite solar cells. This layer was constructed by spin-coating on a polystyrene sphere template. It was demonstrated that the unique structure could enhance the light harvesting efficiency via scattering and promote the crystallinity of the supported perovskite film and the perovskite/TiO_2_ interface, thereby improving the performance of the carbon-perovskite solar cell. The authors achieved 9.81% power conversion efficiency.

## 4. Summary

Photonic crystals have potentially wide applications in photovoltaics and could significantly improve the efficiency of solar cells, as shown in Table 4, which presents the PCE values for examples of SCs with PCs and their percentage increase compared to SCs without PCs.

Photonic crystals can be used as an anti-reflective and light-trapping surface, back reflector, spectrum splitter, absorption enhancer, radiation cooler, or electron transport layer, depending on the kind of solar cell. The purpose of each of these elements is, among others, to redirect, concentrate, or trap incident radiation, which allows for better use of light by the solar cell. Appropriate light management reduces the active material used, which has ecological and economic significance. In addition to photonic crystals as light-trapping systems, research is being conducted on other nanostructures that control and manipulate light behavior, such as plasmonic absorbers [191,192,193]. Plasmonic absorbers also consist of micro- and nanostructured materials that can interact with light at the subwavelength scale to obtain unique optical properties. Still, unlike photonic crystals, they exploit the plasmonic properties of metallic nanostructures.

In addition, photonic crystals, due to their structural color that is a consequence of their periodic structures, can also improve the appearance of solar cells, which is of great importance when SCs are incorporated into various indoor and outdoor architectural designs. Almost all kinds of photonic crystal structures are considered in solar cells of all generations, mostly only theoretically. Not all the analyzed structures are easy to use for technological reasons and often require advanced nanofabrication techniques. In general, the production of photonic crystals is more cost-intensive the greater the dimensionality of the PCs. Hence, proposals for photonic crystals implemented in solar cells are mostly limited to 1D and 2D structures. In the case of 3D structures, only inverse opal structures resulting from relatively cheap technology based on self-assembly are taken into account [189]. In the literature, the following techniques to produce SC prototypes are proposed: chemical vapor deposition [179], physical vapor deposition [103], spin coating [187], reactive-ion etching (RIE) [12], nano-imprinting techniques [184], and atomic layer deposition (ALD) [189]. However, the balance of production costs and benefits should be considered. Developing effective technology is still one of the most severe challenges. Implementing photonic crystals in SCs is a challenge in technology development for the production of PCs and their integration with the SCs. The next significant engineering challenge is optimizing the design and geometry of photonic crystals to minimize optical losses while maximizing light absorption and achieving broad-band light trapping and absorption across the solar spectrum while minimizing spectral dependencies. These challenges require a thorough understanding of the crystal lattice’s interaction with light and matter. Photonic crystals fabricated using nanostructured materials may be susceptible to degradation, environmental factors, and long-term stability issues. Ensuring the durability and stability of photonic crystal-enhanced solar cells under real-world operating conditions is crucial for commercial viability. Developing materials and techniques that protect photonic crystals from degradation and environmental factors is essential for long-term device performance. Addressing these challenges requires interdisciplinary research efforts spanning materials science, nanotechnology, photonics, and device engineering. Despite this, reports on the commercialization of technological solutions using photonic crystals in solar cells, e.g., ref. [194], are already available.

## Figures and Tables

**Table 2:** Details of 1D PCs theoretically analyzed as ARC.

Reference	Type	No. of Periods	Materials	Optimum Thickness	Solar Cell
[26]	Ternary	1	SiO_2_/Si_3_N_3_/SiO_2_	98 nm/48 nm/8 nm	PIN silicon
[74]	Ternary	1	SiO_2_/KBr/SiO_2_	98 nm/48 nm/8 nm	PIN silicon
[75]	Binary	1 or 2	Si_x_O_y_N_z_		a-Si:H
[71]	Binary	1	SiO_2_/TiO_2_	54 nm/82 nm	a-Si
[71]	Ternary	1	SiO_2_/Si_3_N_3_/TiO_2_	54 nm/33 nm/82 nm	a-Si
[69]	Ternary	1	SiO_2_/Al:ZnO/SiO_2_	98 nm/48 nm/8 nm	a-Si:H
[69]	Ternary	1	SiO_2_/SiON/SiO_2_	98 nm/48 nm/8 nm	a-Si:H
[76]	Binary		MgF_2_/ZnS	92.39 nm/54.95 nm 92.39 nm/109.91 nm	
[76]	Ternary		MgF_2_/ZnS/Al_2_O_3_	92.39 nm/109.91 nm/ 78.70 nm	

**Table 3:** Details of 2D PCs used as anti-reflective and light-trapping surfaces.

Reference	Type of 2D PC	Lattice	Solar Cell	Methodology
[77]	Tapered silicon	Hexagonal a = 375 nm		Theoretical and experimental
[78]	Round pillars Cones Round pillars Square pillars Inverted pyramids Round holes Round holes Square holes	Square Square Hexagonal Rectangular Square Square Hexagonal Square	On <100> silicon	Theoretical and experimental
[82]	Holes in ITO/p-AlGaAs layer	Hexagonal	GaAs	Theoretical
[79]	Nanocylinders of Al_2_O_3_ or PMMA	Square a = 440 nm	GaAs Si	Theoretical
[80]	Inverted nanopyramid surface texture		Thin-film c-Si	Theoretical and experimental
[81]	Teepee-like PC	a = 1200 nm	c-Si	
[83]	Parabolic-pore thin-c-Si inverted pyramid	a = 1000 nm	Thin-film c-Si	Theoretical
[84]	Inverted micropyramid surface texture	a = 1300 nm, 1800 nm, 2100 nm, and 2500 nm,	Thin-film c-Si	Theoretical

**Table 4:** The values of power conversion efficiency (PCE) and its increase (ΔPCE) as a result of the use of PCs. For comparison, the values of ΔPCE were calculated according to the formula (PCEPC−PCEr)/PCEr where PCEPC is the efficiency of SCs with PCs and PCEr is the efficiency of reference SCs without PCs. *: value is taken from the reference literature.

Reference	Solar Cell	Type of PC	Function of PC	PCE, %	ΔPCE, %	Methodology
[187]	Perovskite	2D honeycomb-like	Light management	20.85	12.1	Experimental
[83]	c-Si	2D parabolic-pore inverted pyramid	Light trapping	29.11	6.8	Theoretical
[111]	c-Si	3D inverse opal	Back reflectors	30.43	16.0	Theoretical
[109]	Thin film CdTe	2D lattice of rods	Back reflector	25.51	23.1	Theoretical
[103]	CdS/CdTe-based	1D multilayer	Back reflector	10.47	26.7	Experimental
[179]	Polymer	1D multilayer	Back reflector	8.20 5.41	23.3 26.4	Experimental
[188]	Perovskite	2D lattice of rods	Absorption enhancer	20.97	20.9	Theoretical
[189]	Dye-sensitized	3D inverse opal	Absorption enhancer	6.875	21.7	Experimental
[190]	Organic	1D multilayer	RC	8.98	1.5	Theoretical
[190]	Perovskite	1D multilayer	RC	12.5	2.4	Theoretical
[173]	Thin-film c-Si	1D multilayer with the SiO_2_ grating	RC	-	0.45 *	Theoretical
[183]	Hybrid perovskite	3D inverse opal	ETL	13.11	19.2	Theoretical
[184]	Perovskite	2D lattice of hemispheres	ETL	15.2	44.7	Experimental
[12]	Perovskite	2D array of nanodisks	ETL	18.70	19.6	Experimental

## Data Availability

Not applicable. No new data were created or analyzed in this study.
